# Desirable Difficulties in Language Learning? How Talker Variability Impacts Artificial Grammar Learning

**DOI:** 10.1111/lang.12464

**Published:** 2021-07-10

**Authors:** Federica Bulgarelli, Daniel J. Weiss

**Affiliations:** a Duke University and The Pennsylvania State University; b The Pennsylvania State University

**Keywords:** artificial grammar learning, statistical learning, talker variability, desirable difficulties, contextual interference

## Abstract

Contending with talker variability has been found to lead to processing costs but also benefits by focusing learners on invariant properties of the signal, indicating that talker variability acts as a desirable difficulty. That is, talker variability may lead to initial costs followed by long-term benefits for retention and generalization. Adult participants learned an artificial grammar affording learning of multiple components in two experiments varying in difficulty. They learned from one, two, or eight talkers and were tested at three time points. The eight-talker condition did not impact learning. The two-talker condition negatively impacted some aspects of learning, but only under more difficult conditions. Generalization of the grammatical dependency was difficult. Thus, we discovered that high and limited talker variability can differentially impact artificial grammar learning. However, talker variability did not act as a desirable difficulty in the current paradigm as the few evidenced costs were not related to long-term benefits.

## Introduction

During the process of language acquisition, learners must track speech or manual signals in order to infer the underlying generating model. A well-known challenge for hearing learners is that the input is typically produced by a variety of talkers with distinct vocal characteristics, reflecting variables such as gender, topic, and dialect. Because of these distinct vocal characteristics, the same speech category can differ greatly in its realization (see Boland, Kaan, Valdés Kroff, & Wulff, 2016; Peterson & Barney, 1952). How does exposure to these kinds of variability impact learning? Previous research has indicated that talker variability can lead to processing costs for learners across the lifespan (Jusczyk, Pisoni, & Mullennix, 1992; Mullennix, Pisoni, & Martin, 1989; Ryalls & Pisoni, 1997). However, increased talker variability can also be helpful for language learning (e.g., Rost & McMurray, 2009); by focusing learners on invariant properties of the signal across talkers, it may help learners to abstract the core principles of linguistic structure (e.g., Graf Estes, 2012; Houston & Jusczyk, 2000). These discrepant findings suggest that talker variability during learning may act as a desirable difficulty for the learner (Bjork, 1994), initially introducing a challenge for learners to overcome, followed by better retention and generalization.

One method that reliably produces a desirable difficulty is presenting materials in a variable manner, such as by interleaving or spacing trials, which results in temporary interference for the learner (e.g., Bjork, 1994). This pattern has been extensively studied in nonlinguistic domains (e.g., learning motor movements; Shea & Morgan, 1979) and has more recently been investigated in the language domain (Bjork & Kroll, 2015). For example, interleaving five different second language (L2) grammatical structures, compared to blocking presentation of each type, led to the largest number of errors during training but also to higher accuracy on a grammaticality judgment task after a week-long delay (Nakata & Suzuki, 2019). Similarly, spacing between presentations benefited L2 vocabulary learning, specifically for semantically unrelated words (Nakata & Suzuki, 2019; see also Suzuki, Nakata, & DeKeyser, 2019; though see Suzuki, 2017 for evidence that shorter [3.3 days] spacing may be more desirable than longer [7 days] spacing for simple and complex morphological features).

Here we ask whether including talker variability during training is another way of presenting materials in a variable manner to create a desirable difficulty. Whereas the majority of studies have focused on varying *how* the input is presented to learners (e.g., through interleaved and spaced practice), varying the stimuli themselves may have a similar effect. Supporting this notion, previous research suggests that increasing the number of exemplars in an artificial grammar learning task allows both infant and adult learners to learn a nonadjacent dependency that they are unable to learn when there is less variability in the exemplars (Gómez, 2002).

## Background Literature

Decades of research investigating how learners process talker variability have yielded seemingly conflicting results, suggesting that across tasks and timescales, talker variability can be both helpful and harmful for learning. For example, research on talker normalization (Nusbaum & Magnuson, 1997), the process of extracting the underlying commonalities across speakers, has shown that changes in talker from trial to trial have detrimental effects on word recognition (Creel & Bregman, 2011; Mullennix et al., 1989). Preschool-aged children have greater difficulty identifying familiar words when presented by 10 talkers than when presented by a single talker, although they improve in contending with variability as they get older (Ryalls & Pisoni, 1997). Similarly, adults exhibit lower accuracy for word recognition when words are produced by 15 different talkers than when produced by one talker (Mullennix et al., 1989). In a more recent study, both adult and child native speakers of Greek learning English words from four different talkers exhibited lower accuracy during training compared to those learning the words from a single talker (Giannakopoulou, Brown, Clayards, & Wonnacott, 2017).

However, increased variability can also be beneficial for learning. For example, increased variability can encourage learners to extract invariant properties of the input (Hollich, Jusczyk, & Brent, 2002). Rost and McMurray (2009) found that increased talker variability helped 14-month-old infants learn novel minimal pairs that could not be acquired when the words were produced by a single talker, an effect that has similarly been shown for infants learning novel phonotactic rules (Seidl, Onishi, & Cristia, 2014). Preschoolers also benefit from increased talker variability, as 4-year-olds were faster and more accurate in producing novel words when they were spoken by different talkers during training (Richtsmeier, Gerken, Goffman, & Hogan, 2009).

For adults, increased talker variability appears to be particularly useful in L2 learning contexts. For example, training from multiple talkers can help L2 learners acquire a difficult phonemic contrast and generalize it to novel talkers (Lively, Logan, & Pisoni, 1993; Logan, Lively, & Pisoni, 1991). Further, multi-talker stimuli facilitate the acquisition and generalization of lexical tones by L2 learners even in the absence of experimenter feedback (Liu & Zhang, 2016). Talker variability has also been found to help adult learners acquire L2 vocabulary (e.g., Barcroft & Sommers, 2005; Sinkeviciute, Brown, Brekelmans, & Wonnacott, 2019). Together, these results suggest that although high talker variability may be challenging for speech processing, it can also be beneficial for multiple aspects of language learning, for both infants, young children, and adults.

The research reviewed thus far focused on the costs and benefits associated with high talker variability (i.e., input from many talkers). Limited talker variability, which we define as two talkers, has also been found to produce helpful and detrimental effects for learners, depending on the experimental task. Limited talker variability is thought to encourage learners to track talker-specific information, conflating talker and linguistic information in the initial stages of processing (Goldinger, 1998). That is, learners may come to associate specific linguistic patterns (e.g., phonotactic or grammatical patterns; see Kamide, 2012; Weatherhead & White, 2016) with specific talkers. This can lead to an advantage in processing speed that results from hearing familiar talkers (e.g., Nygaard & Pisoni, 1998) or from hearing a talker produce the same words across time (e.g., Goldinger, 1998; Palmeri, Goldinger, & Pisoni, 1993). In fact, limited talker variability seems to be particularly useful for learning talker-specific information (see also Choi & Shukla, 2021). For example, when learners are presented with multiple underlying regularities (i.e., artificial speech streams), a change in talker that happens in tandem with the change in regularity helps learners separately track each set of regularity statistics, which they are unable to do when both speech streams (with the different regularities) are produced by the same talker (Antovich & Graf Estes, 2018; Gonzales, Gerken, & Gómez, 2018; Weiss, Gerfen, & Mitchel, 2009).

As noted, conflating linguistic and talker-specific information can also interfere with learning. This is particularly true when learners erroneously track talker-specific information instead of integrating regularities across talkers. For instance, when infant learners were presented with a single artificial speech stream presented by two talkers, they did not exhibit learning, possibly because they were tracking talker-specific information rather than aggregating statistical regularities across talkers (Graf Estes & Lew-Williams, 2015; see also Hollich et al., 2002). Adult L2 learners exhibited lower accuracy in identifying stress patterns produced by two talkers compared to those produced by a single talker, suggesting that they may have encoded the stress patterns as talker-specific rather than abstracting regularities across talkers (Schwab & Dellwo, 2017). Together, the findings indicate that limited talker variability can be beneficial when each talker is correlated with a specific linguistic pattern and talker-specific information is informative. Conversely, limited variability appears to be detrimental for identifying regularities across talkers.

In sum, a case can be made that both high and limited talker variability can pose challenges (e.g., Graf Estes & Lew-Williams, 2015; Houston & Jusczyk, 2000; Mullennix et al., 1989; Ryalls & Pisoni, 1997) and be beneficial for learning (e.g., Clopper & Pisoni, 2004; Rost & McMurray, 2009). However, these costs and benefits have been observed across different tasks, spanning word recognition and learning, acquiring phonotactic or stress patterns, and extracting statistical regularities. Consequently, it is difficult to infer whether the costs may be related to downstream benefits. Therefore, our study sought to determine whether varying the input by introducing talker variability (high or limited) could act as a desirable difficulty, such that initial costs lead to later benefits. Participants were taught an artificial grammar in one of three talker variability conditions: a one-talker condition (i.e., no talker variability), a two-talker condition (i.e., limited talker variability), and an eight-talker condition (i.e., high talker variability).

## The Present Study

Because the costs and benefits of talker variability may only be evident for some components of learning (e.g., word learning or abstracting regularities), we adopted an artificial grammar learning paradigm that involved grammatical gender and spanned multiple aspects of learning. Our task was based on that of Arnon and Ramscar (2012), who presented participants with novel labels for familiar objects. The objects were divided into two categories, each of which was assigned an article to mirror grammatical gender without phonological or semantic cues to signal class membership. Learners acquired both the noun labels and the article–noun pairings (although learning of the latter depended on training order; see Arnon & Ramscar, 2012), with nouns learned better than the article–noun pairs. In the present study, in addition to teaching novel object labels and article–noun pairings, we added a phonological cue to the nouns to signal category membership, allowing us to test generalization to novel stimuli that conformed to the phonological pattern. To test our prediction that talker variability may be particularly useful for learning novel contrasts (as in, e.g., Lively et al., 1993; Rost & McMurray, 2009), we varied the phonological cue across two different experiments. The cue used in Experiment 1 was rather subtle, whereas the one used in Experiment 2 was more overt in an effort to simplify the task.

Because desirable difficulties can only be evidenced by testing at multiple time points, participants were tested on all three components of the artificial grammar task (learning object labels, learning article–noun pairings, and generalizing to novel stimuli) at three time points over the course of a week. Testing at multiple time points further allows for insight into patterns of retention for multiple components of an artificial grammar. We also included a working memory task (N-back, based on Hakun & Johnson, 2017), allowing us to investigate whether differences in performance were related to differences in working memory capacity, as other studies have found that individual differences in perceptual aptitude can influence whether learners benefit from talker variability (Dong, Clayards, Brown, & Wonnacott, 2019; Perrachione, Lee, Ha, & Wong, 2011). Given that bilingual experience has been shown to impact how learners process input from different talkers (e.g., Bregman & Creel, 2014), we restricted our study to monolingual adults (who only had experience of some foreign language education later in life), in an effort to attain a homogeneous sample with respect to language background.

Thus, in the current study we aimed to address the following research questions:

1. To what extent do high and limited talker variability during artificial grammar learning act as desirable difficulties, as evidenced by lower initial accuracy but better long-term retention and generalization?
2. What are the patterns of retention across the three components of artificialgrammar learning (learning object labels, learning article–noun pairings, and generalizing to novel stimuli)?

## Experiment 1

### Method

#### Participants

Participants were 144 monolingual American English speakers (20 males; *M*_age_ = 18.85 years, *SD* = 1.50) who were recruited from an Introduction to Psychology course and received course credit for participation. We assigned 48 participants to each condition. Due to a foreign language requirement, all but one participant reported experience of learning another language, although they self-rated their proficiency at 3.36 (*SD* = 1.34) on a 10-point scale, and none self-rated above 6 (self-rating above a 6 is a criterion previously used to identify bilingual participants; Poepsel & Weiss, 2016). For the majority of participants (133 of 144), the foreign language with which they reported experience was one with grammatical gender (Spanish, French, or Italian). An additional 59 participants were recruited but excluded due to not returning for the delayed test (*n* = 19), reporting high proficiency in a language other than English or having learned a language other than English prior to the age of 8 (*n* = 14), not following instructions (*n* = 7), technical difficulties (*n* = 7), or reporting low effort (self-reporting 5 or below on a 10-point scale; *n* = 12; see Mitchel & Weiss, 2010, 2014 for a similar approach and cut-off criterion)^1^. We obtained informed consent from all participants prior to participation.

### Stimuli

All stimuli (Bulgarelli & Weiss, 2021a) are available on both IRIS (https://iris-database.org) and OSF (https://osf.io/zrmqh/). The stimuli consisted of 60 novel objects, 60 novel nouns, and two novel articles adapted from previous experiments (Arnon & Ramscar, 2012; Poepsel & Weiss, 2016; see Appendix S1 in the Supporting Information online). Twenty-four of the novel object–noun pairs were used during familiarization, with the remainder used at test. The objects consisted of black and white complex line drawings. Eight objects appeared in the stimuli used by Creel, Aslin, and Tanenhaus (2008) and served as a template for creating the remaining objects (using MS Paint^©^). The objects were converted to a .jpeg file format with a size of 150 × 150 pixels. All of these objects had been used in a previous experiment (Poepsel & Weiss, 2016). All of the novel nouns were created using English phonology and ranged from two to three syllables, ending with a vowel. All final syllables were CV, whereas syllables that occurred earlier in the word could be CV, CVC, or CCV. Half of the nouns ended in a front /a/ vowel (Category 1), and the other half ended with a back /ɑ/ vowel (Category 2). The articles were two monosyllabic artificial words (for English speakers), *sem* and *bol*, which had been used in a previous study (Arnon & Ramscar, 2012).

The auditory stimuli were recorded by eight female native speakers of American English who were asked to produce the vowel contrast. We recorded each word and article in a sentence frame. Each target word (article or noun) was spliced from the sentence frame in which it occurred in its correct position: Each noun was recorded in sentence-final position (e.g., “This is a barcha”), and each article was recorded in sentence-initial position (e.g., “Bol apple”). This was done to ensure consistency in pronunciation across conditions. The trained talkers were recorded in a soundproof booth using a Dynamic^©^ microphone connected to a Marantz^©^ recorder. The procedure for the recordings was as follows: The first trained talker (i.e., the same person each time) read a sentence out loud, and then each talker was asked to repeat that sentence. This was done to ensure that phonetic features (such as vowels and flaps) remained relatively consistent across talkers.

Background noise was removed from the recordings using the Noise Reduction feature in Audacity^©^. Each word was subsequently normalized to a maximum amplitude using the Normalization feature in Audacity^©^, such that peak volume remained consistent across all talkers. The articles and nouns were concatenated to form noun phrases (e.g., “Sem barcha”). The noun phrases were created such that the article was always perfectly correlated (i.e., occurred with) with one of the two categories of nouns, and the article–category pair was counterbalanced across participants. This resulted in an underlying regularity between the article and the vowel ending of the nouns, which we refer to as the category rule.

### Design

We assigned each participant to one of three conditions: one-talker, two-talker, and eight-talker. In the one-talker condition, all stimuli were presented by a single talker (counterbalanced between two talkers) and hence did not have between-talker variability. In the two-talker condition, all stimuli were presented by two talkers (the same two talkers that were counterbalanced between participants for the one-talker condition); participants heard each item produced by each talker. In the eight-talker condition, stimuli were presented by all eight talkers, resulting in high between-talker variability during familiarization; as each item was repeated five times, not all talkers produced all items, but each instance of an item was produced by a different talker. For trials in the two-talker and eight-talker conditions, the talker varied randomly trial by trial (i.e., familiarization was not blocked by talker). Participants heard a single talker at test (one of the two used in the one- and two-talker conditions); this was counterbalanced across participants, and the talker was always one heard during familiarization (see Appendix S2 in the Supporting Information online for counterbalancing and Appendix S3 there for the mean pitch of experimental talkers).

### Procedure

**Figure 1:**
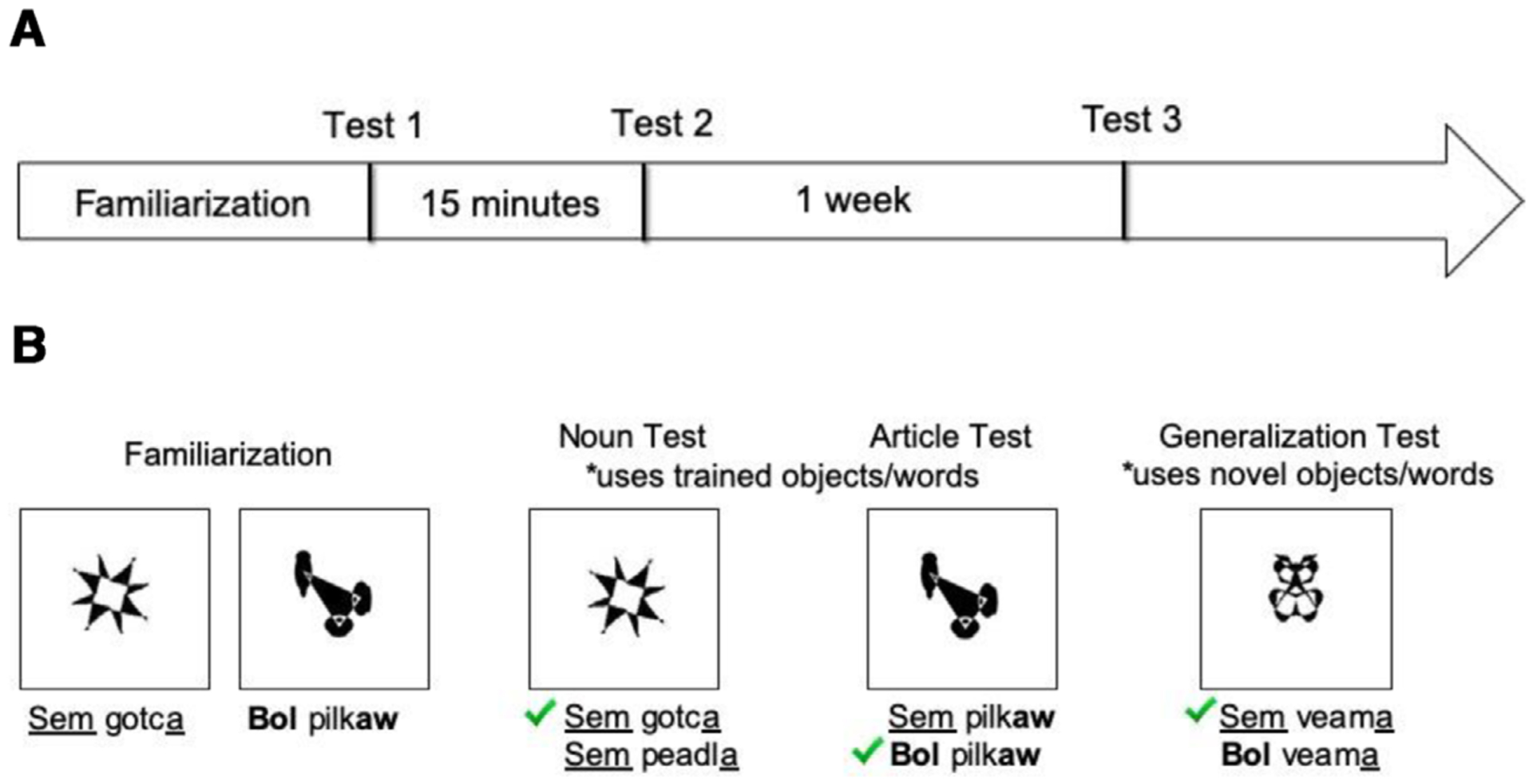
Details of the experiment: Panel A: Timeline and Panel B: Examples of familiarization and test trials.

Participants were seated at a computer in a sound-attenuated booth and instructed that they would learn novel names for novel objects. They were asked to listen through headphones and told that they would be asked questions about what they learned. During familiarization, participants viewed a picture of one of the objects on the screen and heard a noun phrase describing the object. Each of the 24 objects used during familiarization was repeated five times, in randomized order, for a total of 120 trials. Each image remained on the screen for 5 seconds, and then the program automatically advanced to the next image and corresponding noun phrase. Following familiarization, participants completed three test types (described below) that were conducted at three time points (see Figure 1A). The first test (immediate test) occurred immediately after familiarization, and the second test occurred 15 minutes later (short-delay test). During the 15-minute delay, participants completed an N-back task of working memory and a Language History Questionnaire (Li, Sepanski, & Zhao, 2006). Participants subsequently returned a week later to complete the third test (long-delay test).

Each test type consisted of 12 test items, totaling 36 items per test phase. For all of the tests, participants viewed a single image on the screen and heard two noun phrases, separated by a 1-second pause. Participants were then asked to choose which noun phrase correctly described the image (by pressing “1” for the first option or “2” for the second). The image remained on the screen until the participant made a response. The order of the sentences heard during all tests was counterbalanced across trials, such that half the trials presented the correct sentence first, and half the incorrect sentence first.

The first test type asked whether participants had learned the noun–object mappings (hereafter “Noun Test”). During the Noun Test, participants always heard noun phrases consisting of the correct article paired with either the correct or an incorrect noun (see Figure 1B). The competitor noun was always from the same category, so that it occurred with the same article during familiarization. Twelve of the object–noun pairs (six per category) from familiarization were used for the Noun Test across all testing phases.

The second test type (Article Test) investigated learning of the article–noun pairings. Participants heard two noun phrases consisting of the correct noun and either the correct or incorrect article. The 12 object–noun pairs from familiarization not used for the Noun Test were used for the Article Test across all testing phases.

The third test type assessed participants’ ability to generalize the category rule to novel instances (Generalization Test). Participants heard phrases containing a single novel label that matched one of the category rules (e.g., *veama* matched Category 1, and *bosaw* matched Category 2). One of the noun phrases contained the article *sem*, and the other noun phrase contained the article *bol* (e.g., *Sem veama* vs. *Bol veama*; see Figure 1B). Participants had not previously encountered the object or label, but if they inferred the category rule, they should select the correct noun phrase based on the article correctly matching the phonological category of the word. The 36 novel objects and labels not used during familiarization were used for the Generalization Test, 12 at each of the three testing phases. The Generalization Test was very similar to the Article Test, the critical difference being that in the Article Test, participants responded to article and noun pairs that they had heard during familiarization, whereas in the Generalization Test, the nouns and objects were novel. In order to succeed on the Generalization Test, participants had to acquire and generalize the category rule, whereas for the Article Test they could respond correctly by either learning the rule or recalling instances of article + noun combinations from familiarization. During the experiment, for each test phase, participants completed the Generalization Test first, to minimize additional exposure to the familiarized items before generalization, followed by the Noun Test and then the Article Test.^2^

### Working Memory

Participants also completed the N-back test of working memory, consisting of four conditions (adapted from Hakun & Johnson, 2017). In the compare condition, participants judged whether two letters, presented side by side, were the same. In all other conditions (1-back, 2-back, and 3-back), participants viewed one letter at a time for 2,000 milliseconds each, followed by a 500-millisecond interstimulus interval before the next letter. During the 1-back condition, participants were asked to judge whether the current letter was the same as the letter presented immediately before it. For the 2-back and 3-back conditions, participants had to judge whether the current letter matched the letter presented two or three letters before it, respectively. The letters used during the task were B, F, K, H, M, Q, R, and Y. Each block type (1-back, 2-back, and 3-back) consisted of 15 trials, with four trials per block requiring a “yes” response. Overall accuracy was calculated for each participant by averaging performance on the 1-back, 2-back, and 3-back conditions. For each condition, trials at the beginning of the block that could not have been responded to according to the rule were excluded (e.g., the first trial in the 1-back condition, as no trial occurred before it). Internal consistency (Cronbach’s alpha) for this N-back task ranged from .86 (Experiment 1) to .89 (Experiment 2).

## Results

We performed all analyses in R (R Studio, 2019) using the lme4 package (Bates, Mächler, Bolker, & Walker, 2015); all data (Bulgarelli & Weiss, 2021b) and analyses scripts (Bulgarelli & Weiss, 2021c) can be found on both IRIS (https://iris-database.org) and the OSF (https://osf.io/zrmqh/). We conducted three separate models, one each for the Noun, Article, and Generalization Tests. We carried out a logistic mixed-effects analysis investigating performance across the immediate, short-delay, and long-delay tests based on condition. Test time (immediate, short delay, and long delay), talker variability condition (one-talker, two-talker, and eight-talker), and the interaction between these variables were fixed effects; N-back performance and talker used at test were entered as covariates; and by-participant and by-item random effects were added to the model.^3^ The contrasts for talker variability conditions tested the effect of talker variability compared to no variability (contrast coding: one-talker = 1, two-talker = −.5, eight-talker = −.5) and then compared the two talker variability conditions to each other (contrast coding: one-talker = 0, two-talker = 1, eight-talker = −1). The contrasts for test time addressed our second research question, testing differences between the immediate and short-delay tests (contrast coding: immediate = 1, short delay = −1, long delay = 0) and comparing the immediate and short-delay tests to the long-delay test (contrast coding: immediate = −.5, short delay = −.5, long delay = 1). The interactions of these two contrasts indicated whether patterns of retention or forgetting differed across talker variability conditions, addressing our first and second research questions.

The final model formula in R was: Accuracy ~ TalkerVariabilityCondition * Test time + N-back + Test Talker + (1 + TalkerVariabilityCondition|Subject) + (1|Item).^4^

**Figure 2:**
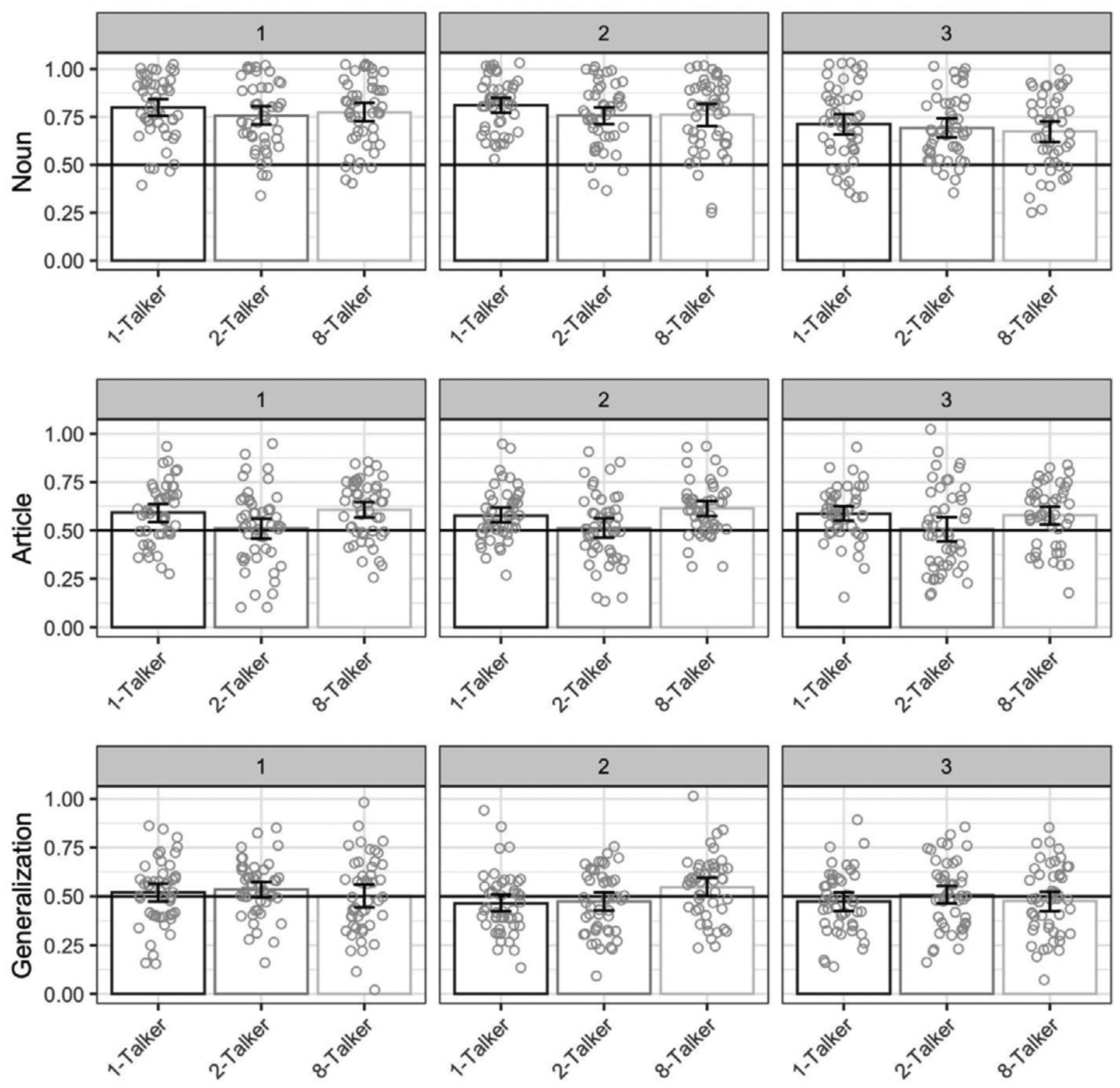
Experiment 1 raw mean accuracy (*y*-axis), across test time (panels labeled 1, 2, and 3), by condition (on *x*-axis). The test types (Noun, Article, Generalization) are each plotted separately. Error bars reflect 95% confidence intervals. The black lines indicate chance performance (50%).

Visual inspection of residual plots did not reveal any obvious deviations from normality or homoscedasticity. Raw performance across all test types, test times, and conditions can be found in Figure 2 and Table 1, along with estimated marginal means and 95% confidence intervals from the models. Throughout the manuscript, alpha is set at .05; Bonferroni corrected alpha is .006 (based on correcting for nine comparisons). See Appendix S4 in the Supporting Information online for full model tables.

### Noun Test

As Table 1 shows, performance on the Noun Test exceeded chance across all test times and conditions even after the Bonferroni correction for multiple comparisons was applied. The logistic mixed-effects model for the Noun Test accounted for a small to medium amount of variance (marginal *R*^2^ = .03, conditional *R*^2^ = .26). The model revealed an effect of test time, such that performance at the immediate test and the short-delay test (*M* = 78, *SD* = 17) was significantly higher than at the long-delay test (*M* = 69, *SD* = 19), *b* = −0.52, *SE* = 0.07, 95% CI [−.66, −.38], *z* = 7.19, *p <* .001. There was no difference in performance between the immediate and the short-delay test, *b* = −0.005, *SE* = 0.09, 95% CI [−.18, .17], *z* = −0.06, *p* = .95, suggesting that forgetting occurred only after the longer delay. Performance did not significantly differ between the one-talker condition and the two- and eight-talker conditions, *b* = 0.20, *SE* = 0.18, 95% CI [−.15, .56], *z* = 1.13, *p* = .26, or between the two-talker and eight-talker conditions, *b* = −0.05, *SE* = 0.22, 95% CI [−.47, .38], *z* = 0.22, *p* = .83, indicating that talker variability condition did not impact word learning. The effect of the talker used in testing was not significant, *b* = 0.14, *SE* = 0.17, 95% CI [−.20, .48], *z* = 0.83, *p* = .41.

None of the interactions was significant (Immediate vs. Short Delay × One-Talker vs. Two- and Eight-Talker, *b* = −0.10, *SE* = 0.19, 95% CI [−.48, .27], *z* = −0.55, *p* = .58; Immediate vs. Short Delay × Two-Talker vs. Eight-Talker, *b* = −0.10, *SE* = 0.21, 95% CI [−.52, .31], *z* = 0.49, *p* = .62; Immediate and Short Delay vs. Long Delay × One-Talker vs. Two- and Eight-Talker, *b* = −0.12, *SE* = 0.15, 95% CI [−.42, .18], *z* = 0.76, *p* = .45; Immediate and Short Delay × Two-Talker vs. Eight-Talker, *b* = 0.17, *SE* = 0.17, 95% CI [−.17, .51], *z* = 0.98, *p* = .33). There was, however, an effect of N-back performance, *b* = 1.75, *SE* = 0.60, 95% CI [.58, 1.91], *z* = 2.93, *p* = .003, such that better performance on the working memory task was correlated with higher accuracy on the Noun Test, *r* = .08, 95% CI [.06, .11], *p <* .001.

### Article Test

As Table 1 shows, participants in the two-talker condition never exceeded chance in the Article Test, whereas those in the one- and eight-talker conditions did so at all three time points. The logistic mixed-effects model for the Article Test accounted for a small amount of variance (marginal *R*^2^ = .009, conditional *R*^2^ = .07). This model revealed no effects of test time, such that performance did not change from the immediate to the short-delay test, *b* = −0.007, *SE* = 0.071, 95% CI [−.13, .15], *z* = −0.1, *p* = .92, nor from the immediate and short-delay tests to the long-delay test, *b* = −0.04, *SE* = 0.06, 95% CI [−.16, .08] *z* = −0.68, *p* = .50, suggesting that participants who performed above chance on the immediate test did not forget the article–noun mappings over the course of the experiment. Whereas performance did not differ between the one-talker condition and the two talker variability conditions, *b* = 0.12, *SE* = 0.08, 95% CI [−.06, .31], *z* = 1.43, *p* = .15, performance did differ between the two- and eight-talker conditions, *b* = −0.38, *SE* = 0.12, 95% CI [−.60, −.17], *z* = 3.04, *p* = .002: Participants in the two-talker condition (*M* = 51, *SD* = 20) exhibited lower accuracy than those in the eight-talker condition (*M* = 60, *SD* = 15; see Table 1). The effect of the talker used in testing was not significant, *b* = −0.017, *SE* = 0.08, 95% CI [−.18, .17], *z* = −0.21, *p* = .83, and the effect of N-back was not significant, *b* = 0.31, *SE* = 0.29, 95% CI [−.21, .99], *z* = 1.08, *p* = .28.

None of the interactions was significant (Immediate vs. Short Delay × One-Talker vs. Two- and Eight-Talker, *b* = 0.09, *SE* = 0.15, 95% CI [−.20, .38], *z* = −0.60, *p* = .55; Immediate vs. Short Delay × Two-Talker vs. Eight-Talker, *b* = −0.03, *SE* = 0.18, 95% CI [−.31, .37], *z* = 0.17, *p* = .86; Immediate and Short Delay vs. Long Delay × One-Talker vs. Two- and Eight-Talker, *b* = 0.08, *SE* = 0.13, 95% CI [−.18, .33], *z* = 0.60, *p* = .55; Immediate and Short Delay × Two-Talker vs. Eight-Talker, *b* = 0.14, *SE* = 0.15, 95% CI [−.15, .44], *z* = 0.94, *p* = .35).

### Generalization Test

As Table 1 shows, participants did not exceed chance in the Generalization Test at any test time, regardless of condition after the Bonferroni correction for multiple comparisons was applied. The logistic mixed-effects model for the Generalization Test accounted for a small amount of variance (marginal *R*^2^ = .005, conditional *R*^2^ = .008). This model revealed no effect of test time, such that performance did not change from the immediate to the short-delay test, *b* = 0.09, *SE* = 0.07, 95% CI [−.04, .23], *z* = 1.35, *p* = .18, nor from the immediate and short-delay tests to the long-delay test, *b* = −0.07, *SE* = 0.06, 95% CI [−.19, .04], *z* = 1.23, *p* = .22. There was also no effect of condition, such that performance did not differ between the one-talker and the talker variability conditions, *b* = −0.09, *SE* = 0.06, 95% CI [−.21, .03], *z* = 1.43, *p* = .15, nor between the two-talker and the eight-talker conditions, *b* = −0.008, *SE* = 0.07, 95% CI, *z* = 0.12, *p* = .91.

There was, however, a significant interaction between the two-talker and eight-talker conditions, and the immediate and short-delay tests, *b* = −0.41, *SE* = 0.17, 95% CI [.08, .74], *z* = 2.44, *p* = .015, such that for the two-talker condition, performance decreased from the immediate test (*M* = 54, *SD* = 14) to the short-delay test (*M* = 47, *SD* = 16), whereas for the eight-talker condition, performance increased from the immediate test (*M* = 50, *SD* = 20) to the short-delay test (*M* = 55, *SD* = 16; see Table 1). None of the raw mean comparisons exceeded chance after the Bonferroni correction for multiple comparisons was applied, so we do not interpret this interaction further. All other interactions were not significant (Immediate vs. Short Delay × One-Talker vs. Two- and Eight-Talker, *b* = 0.21, *SE* = 0.15, 95% CI [−.08, .49], *z* = 1.43, *p* = .15; Immediate and Short Delay vs. Long Delay × One-Talker vs. Two- and Eight-Talker, *b* = −0.0002, *SE* = 0.13, 95% CI [−.25, .25], *z* = −0.002, *p* = .99; Immediate and Short Delay × Two-Talker vs. Eight-Talker, *b* = 0.25, *SE* = 0.15, 95% CI [−.04, .53], *z* = 1.69, *p* = .09). The effects of N-back, *b* = 0.33, *SE* = 0.21, 95% CI [−.06, .73], *z* = 1.45, *p* = .10, and talker used at test, *b* = 0.05, *SE* = 0.06, 95% CI [−.06, .17], *z* = 0.88, *p* = .38, were not significant.

## Discussion

We investigated whether talker variability during learning of an artificial grammar acted as a desirable difficulty. Participants were taught an artificial grammar that afforded the learning of three components—nouns that labeled objects, article–noun pairings, and generalization of a category rule—and tested at three time points. Talker variability did not impact learning of the object labels, as performance did not differ across talker variability conditions. By contrast, limited talker variability did impact learning the article–noun pairs, as participants in the two-talker condition did not exhibit learning at any time point, whereas those in the one-talker and eight-talker conditions exceeded chance at all time points. Lastly, talker variability did not impact generalization to novel exemplars, as participants did not exceed chance regardless of talker variability condition. Across the board, retention was robust and not dependent on talker variability condition.

The lack of generalization could suggest that participants learned only the specific token and article combinations due to not being able to hear the vowel contrast, which is not contrastive in American English (the dialect of the participants in the experiment). In order to address this possibility, we conducted an additional experiment testing whether a different group of participants could distinguish between the vowels at the end of the words when the words were presented in isolation. Results from this can be found in Appendix S5 in the Supporting Information online and suggest that participants may have been able to hear the vowel difference in this additional experiment and, therefore, also in Experiment 1. If so, lack of generalization in Experiment 1 could suggest that, despite noticing the difference, participants did not notice it as a defining feature of the category. Given the lingering uncertainty about how the difficult contrast impacted performance, in Experiment 2 we explored this issue further by using a more salient vowel contrast.

## Experiment 2

### Method

In Experiment 1, we modified a task previously used by Arnon and Ramscar (2012) but added a subtle phonological cue to category structure, to enable us to test an additional component of learning: generalization of the grammar to novel exemplars. Comparing performance on the article–noun pairs in Experiment 1 to the results reported by Arnon and Ramscar (2012) suggests that accuracy was quite similar, and the addition of the phonological regularity in Experiment 1 did not appear to impact performance. It is possible that the phonological cue may have been too difficult (although participants could hear the vowel difference; see Appendix S5 in the Supporting Information online), as overall accuracy on the test of generalization was low. In Experiment 2, we investigated whether an easier vowel contrast (/i/ vs. /ɑ/) would lead to better learning of the category rule, as reflected by better performance on the tests of the article–noun pairs and, perhaps, also generalization. The methods were identical to those described previously except for the easier vowel distinction.

### Participants

Participants were 147 L1 American English speakers (23 males; *M*_age_ = 18.43 years, *SD* = 0.71) who were recruited from an Introduction to Psychology course and received course credit for participation. We assigned 48 participants to the one-talker condition, 47 to the two-talker condition, and 52 to the eight-talker condition. Due to a foreign language requirement of their program, all but two participants reported experience of learning another language, although they self-rated their proficiency at 3.4 (*SD* = 1.36) on a 10-point scale, and none self-rated above 6 (as noted for Experiment 1, self-rating above a 6 is a criterion previously used to identify bilingual participants; Poepsel & Weiss, 2016). The majority (130 of 147) reported exposure to a language that has grammatical gender (Spanish, French, or Italian). An additional 83 participants were recruited but excluded due to their not returning for the delayed test (*n* = 27), reporting high proficiency in a language other than English or having learned a language other than English prior to the age of 8 years (*n* = 25), having technical difficulties (*n* = 10), not following directions (*n* = 2), or reporting low-effort (self-reporting 5 or below on a 10-point scale on either day; *n* = 19; see Mitchel & Weiss, 2010, 2014 for a similar approach and cutoff criterion).^5^

### Stimuli

The stimuli were the same as those used in Experiment 1, but now half of the words ended in an /i/ vowel, and the other half ended with a back /ɑ/ vowel (Category 2). The stimuli were recorded by the same eight talkers used in Experiment 1 (see Appendix S3 in the Supporting Information online for mean pitch across experimental talkers).

### Procedure

The procedures and conditions were identical to those used in Experiment 1.

## Results

**Figure 3:**
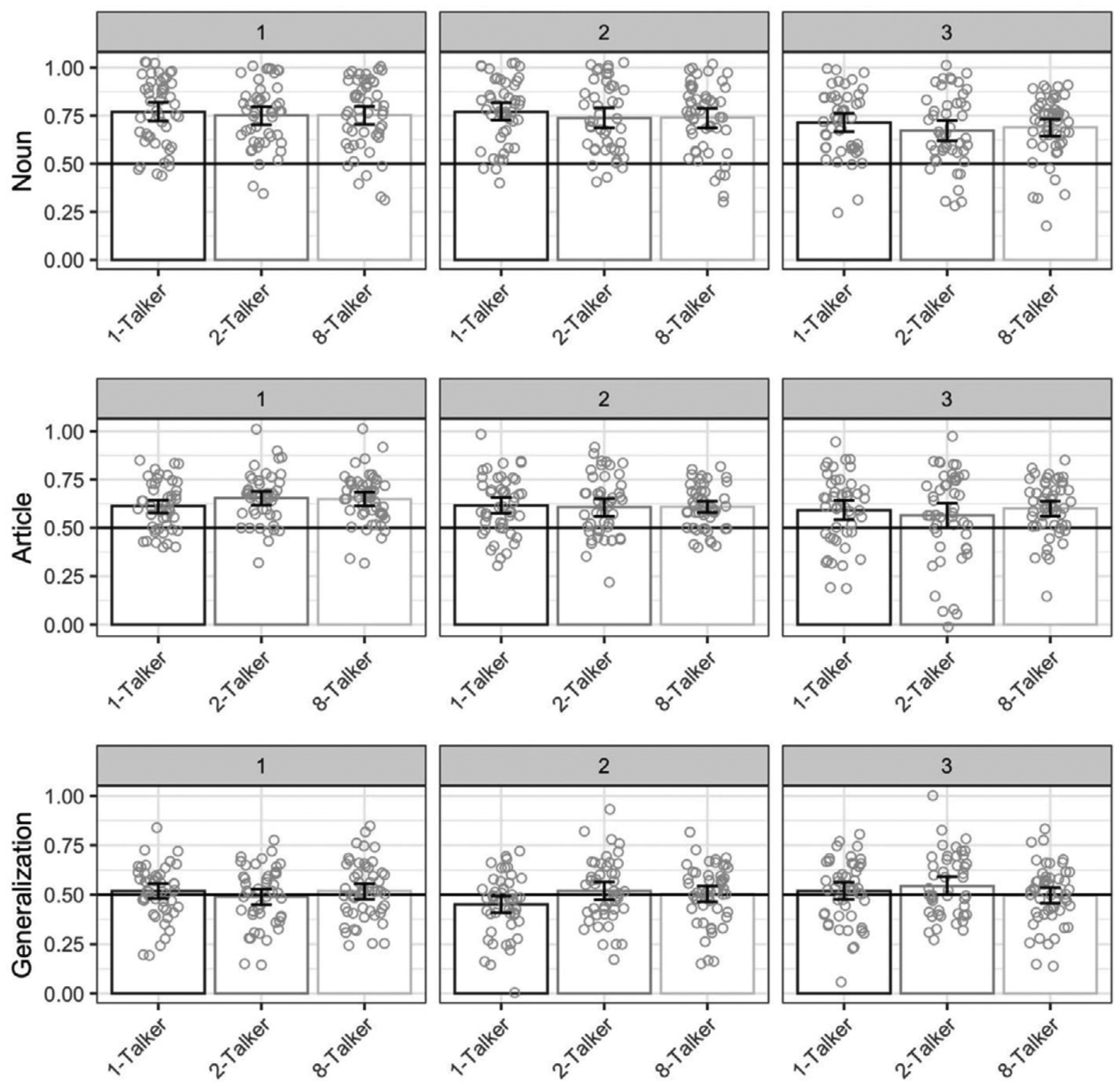
Experiment 2 raw mean accuracy across all three time points, test types, and conditions. Error bars reflect 95% confidence intervals. The black horizontal lines indicate chance performance (50%).

We conducted all analyses in R (R Studio, 2019) using the lme4 package (Bates et al., 2015). Performance for participants in all three conditions can be found in Figure 3 and Table 2.

### Noun Test

Performance on the Noun Test was above chance at all time points, regardless of condition (see Table 2). The logistic mixed-effects model for the Noun Test accounted for a small to medium amount of variance (marginal *R*^2^ = .012, conditional *R*^2^ = .236). This model revealed an effect of test time, such that performance at the immediate test and the short-delay test (*M* = 75, *SD* = 18) was significantly higher than at the long-delay test (*M* = 69, *SD* = 17), *b* = −.36, *SE* = .07, 95% CI [−.49, −.22], *z* = 5.09, *p <* .001. There was no difference in performance between the immediate and the short-delay test, *b* = −0.05, *SE* = 0.08, 95% CI [−.11, .22], *z* = 0.62, *p* = .54, suggesting that forgetting occurred only after the longer delay. Performance did not significantly differ between the one-talker and the talker variability conditions, *b* = 0.12, *SE* = 0.17, 95% CI [−.21, .45], *z* = 0.70, *p* = .48, or between the two-talker and eight-talker conditions, *b* = −0.03, *SE* = 0.20, 95% CI [−.43, .36], *z* = −0.17, *p* = .86, indicating that talker variability condition did not impact word learning. The effects of the talker used at test, *b* = −0.01, *SE* = 0.16, 95% CI [−.33, .32], *z* = −0.36, *p* = .98, and of N-back, *b* = 0.91, *SE* = 0.45, 95% CI [−.04, 1.86], *z* = 1.87, *p* = .06, were not significant.

None of the interactions was significant (Immediate vs. Short Delay × One-Talker vs. Two- and Eight-Talker, *b* = −0.09, *SE* = 0.18, 95% CI [−.45, .26], *z* = −0.52, *p* = .60; Immediate vs. Short Delay × Two-Talker vs. Eight-Talker, *b* = 0.008, *SE* = 0.20, 95% CI [−.39, .40], *z* = 0.04, *p* = .97; Immediate and Short Delay vs. Long Delay × One-Talker vs. Two- and Eight-Talker, *b* = 0.03, *SE* = 0.15, 95% CI [−.26, .33], *z* = 0.22, *p* = .83; Immediate and Short Delay × Two-Talker vs. Eight-Talker, *b* = −0.08, *SE* = 0.17, 95% CI [−.41, .25], *z* = −0.49, *p* = .62).

### Article Test

Participants in all three conditions exhibited above-chance performance on the Article Test at the immediate and short-delay tests, and those in the one- and eight-talker conditions continued to do so even after the long delay, whereas those in the two-talker condition exhibited more forgetting than the other two groups (see Table 2). The logistic mixed-effects model for the Article Test accounted for a small amount of variance (marginal *R*^2^ = .004, conditional *R*^2^ = .051). This model revealed an effect of test time, such that performance on the immediate test and the short-delay test (*M* = 63, *SD* = 13) was significantly higher than on the long-delay test (*M* = 59, *SD* = 18), *b* = −0.17, *SE* = 0.06, 95% CI [−.29, −.05], *z* = 2.83, *p* = .005. There was no difference in performance between the immediate and the short-delay test, *b* = 0.12, *SE* = 0.07, 95% CI [−.02, .26], *z* = 1.72, *p* = .08, suggesting that forgetting occurred only after the longer delay. Performance did not significantly differ between the one-talker and the talker variability conditions, *b* = −0.05, *SE* = 0.09, 95% CI [−.22, .12], *z* = −0.60, *p* = .55, or between the two-talker and eight-talker conditions, *b* = −0.05, *SE* = 0.10, 95% CI [−.25, .14], *z* = −0.54, *p* = .59, indicating that talker variability condition did not impact learning of the article–noun pairs. The effects of the talker used in testing, *b* = −0.03, *SE* = 0.08, 95% CI [−.13, .19], *z* = 0.42, *p* = .67, and of N-back, *b* = 0.29, *SE* = 0.26, 95% CI [−.21, .79], *z* = 1.13, *p* = .26, were not significant.

None of the interactions was significant (Immediate vs. Short Delay × One-Talker vs. Two- and Eight-Talker, *b* = −0.21, *SE* = 0.15, 95% CI [−.51, .09], *z* = −1.37, *p* = .17; Immediate vs. Short Delay × Two-Talker vs. Eight-Talker, *b* = 0.03, *SE* = 0.17, 95% CI [−.31, .37], *z* = 0.17, *p* = .87; Immediate and Short Delay vs. Long Delay × One-Talker vs. Two- and Eight-Talker, *b* = 0.10, *SE* = 0.13, 95% CI [−.16, .35], *z* = 0.75, *p* = .45; Immediate and Short Delay × Two-Talker vs. Eight-Talker, *b* = −0.16, *SE* = 0.15, 95% CI [−.45, .13], *z* = −1.10, *p* = .27).

### Generalization Test

Performance on the Generalization Test never exceeded chance after the Bonferroni correction for multiple comparisons was applied (see Table 2). The logistic mixed-effects model for the Generalization Test accounted for a small amount of variance (marginal *R*^2^ = .003, conditional *R*^2^ = .004). This model revealed no difference between the immediate and the short-delay tests, *b* = 0.07, *SE* = 0.07, 95% CI [−.06, .20], *z* = 1.06, *p* = .29, and no difference between the immediate and short-delay tests relative to the long-delay test, *b* = 0.08, *SE* = 0 .06, 95% CI [−.04, .19], *z* = −1.32, *p* = .19. There were also no differences between conditions (one-talker vs. two- and eight-talker, *b* = −0.07, *SE* = 0.06, 95% CI [−.18, .05], *z* = −1.13, *p* = .26; two-talker vs. eight-talker, *b* = 0.04, *SE* = 0.07, 95% CI [−.10, .17], *z* = 0.55, *p* = .58). There was, however, a significant interaction between the one-talker and talker variability conditions and the immediate and short-delay tests, *b* = 0.30, *SE* = 0.15, 95% CI [.01, .58], *z* = 2.06, *p* = .04. For the one-talker condition, performance decreased from the immediate test (*M* = 52, *SD* = 14) to the short-delay test (*M* = 45, *SD* = 16), whereas it did not for the talker variability conditions (immediate: *M* = 51, *SD* = 15; short delay: *M* = 51, *SD* = 15; see Table 2). Given that performance did not exceed chance after the Bonferroni correction for multiple comparisons was applied, we do not interpret this interaction further.

None of the other interactions was significant (Immediate vs. Short Delay × Two-Talker vs. Eight-Talker, *b* = −0.17, *SE* = 0.16, 95% CI [−.49, .15], *z* = −1.04, *p* = .30; Immediate and Short Delay vs. Long Delay × One-Talker vs. Two- and Eight-Talker, *b* = 0.10, *SE* = 0.13, 95% CI [−.15, .34], *z* = 0.78, *p* = .44; Immediate and Short Delay × Two-Talker vs. Eight-Talker, *b* = 0.22, *SE* = 0.14, 95% CI [−.06, .50], *z* = −1.57, *p* = .12). The effects of the talker used attest, *b* = −0.03, *SE* = 0.06, 95% CI [−.14, .08], *z* = −0.60, *p* = .55, and of N-back, *b* = 0.18, *SE* = 0.18, 95% CI [−.16, .53], *z* = 1.05, *p* = .30, were not significant.

## Discussion

In Experiment 2, we modified the artificial grammar such that the vowel contrast across the two categories was easier to distinguish. Across all test types, there was no effect of the talker variability condition. Further, even participants in the two-talker condition learned the article–noun pairs, in contrast to Experiment 1, a finding which was probably due to the change in the vowel contrast across experiments. Simplifying the vowel contrast may have resulted in less interference from the two-talker condition (because the categories were clearer), but it did not facilitate the ability to generalize in any of the conditions. Thus, Experiment 2 suggested that when categories were easier to distinguish, limited talker variability did not impose a challenge for learning in the context of this experimental task.

## General Discussion

Across two experiments, we investigated the impact of talker variability on multiple aspects of artificial grammar learning, asking whether talker variability might act as a desirable difficulty for the learner. Participants were taught an artificial language that afforded the learning of three types of information (object labels, article–noun pairs, and a category rule). They were taught by one, two, or eight talkers and then were tested at three time points: immediately after familiarization, after a 15-minute delay, and after a 1-week delay. In Experiment 1, participants exhibited learning and retention of the nouns regardless of talker variability condition, suggesting that changes in the number of talkers did not have a detrimental impact on learning. By contrast, only participants in the one- and eight-talker conditions exhibited learning of the article–noun pairs. In Experiment 2, we used an easier vowel contrast to signal category identity and replicated results on the Noun Test (i.e., similar learning regardless of talker variability) while boosting learning of the article–noun pairs in the two-talker condition (i.e., rendering it similar to the no-variability and higher-variability conditions). Across both experiments, performance on the Generalization Test hovered near chance, suggesting that participants learned the frozen article–noun combinations (item learning) but did not acquire the non-adjacent dependency (regularity learning). We also tested whether performance on the artificial grammar task was related to working memory abilities. We found that working memory predicted learning on the Noun Test, although this effect was only significant in Experiment 1.

### Talker Variability as a Desirable Difficulty

If talker variability acted as a desirable difficulty for artificial grammar learning, we would have expected to see initially lower performance for participants in the talker variability conditions relative to the baseline one-talker condition, followed by benefits for retention and/or generalization to novel exemplars. Whereas previous research suggested that contending with rapid changes in talker negatively impacted initial word recognition even for adults (Mullennix et al., 1989), here we found that high talker variability did not pose an additional challenge for artificial grammar learning. Limited talker variability (two talkers), however, did lead to challenges for learners, but only for learning the article–noun pairs and only in Experiment 1. Given that participants had to integrate the input across the two talkers (as both talkers produced exemplars from both categories), tracking talker-specific information seemed to interfere with learning, as has been shown in previous research (Graf Estes & Lew-Williams, 2015). Overall, however, we did not find any evidence that talker variability is beneficial for learning. Thus, we do not find support for the notion that talker variability acts as a desirable difficulty, at least in the context of our task, as the only evidenced cost did not result in a long-term benefit.

Although talker variability may not act as a desirable difficulty, we discovered that high and limited talker variability differentially impacted learning on the same task, but only when the categories were more difficult to distinguish. That is, the addition of limited talker variability (just two talkers) in Experiment 1 inhibited learning of the articles across all three time points (see also Brown & Gaskell, 2014, for sustained talker-specificity effects). In comparison, when the categories were easier to distinguish (Experiment 2), participants exhibited similar levels of learning regardless of talker variability condition. This pattern of results suggests that when the task is more challenging and just two talkers produce the input, learners may be most likely to track talker-specific information, which in this case interfered with learning because both talkers produced both categories. Thus, limited talker variability may only be *helpful* in challenging situations when it perfectly corresponds to changes in structure. Under these circumstances, previous studies have found it can be used as a learning support, perhaps drawing attention to the talker-specific change (e.g., Antovich, Gluck, Goldman, & Graf Estes, 2020; Graf Estes & Lew-Williams, 2015; Weiss et al., 2009; Zinszer & Weiss, 2012).

A lack of evidence that talker variability acts as a desirable difficulty in the current task does not rule out the possibility that it could do so in other settings. For example, it is possible that challenges associated with processing talker variability may be evident *during* learning rather than during a retrospective test. Additionally, talker variability may act as a desirable difficulty for only some aspects of language learning. For example, benefits associated with high talker variability have been found when learners need to acquire contrastive features of the input in their first or second language (e.g., Lively et al., 1993; Logan et al., 1991; Rost & McMurray, 2009). Given the problem of the lack of invariance in the production of sounds across speakers (described at the outset of this article), it is possible that talker variability may be most likely to act as a desirable difficulty in the learning of phonological properties of language (such as minimal pairs). Future research should continue to investigate whether talker variability acting as a desirable difficulty is a viable proposition for other aspects of language learning.

### Retention of Learning

The current study also established a pattern of retention for learning several components of an artificial language at multiple time points in adults, demonstrating that participants retain learned information after short and (relatively) long delays following a brief familiarization period, without any reexposure between the familiarization phase and the tests. Importantly, patterns of retention were not moderated by talker variability during learning. Previous research has shown that adults can retain names of novel objects learned in a cross-situational statistical learning task (i.e., learning form-meaning mappings based on statistical regularities experienced in different contexts, see Walker, Monaghan, Schoetensack, & Rebuschat, 2020 for a recent example) after a week-long delay (Vlach & Sandhofer, 2014). In our study, participants retained both the novel object labels and the article–noun pairs (when they were learned) after 1 week. These results add to a growing body of literature indicating that information learned during artificial language studies can lead to lasting memory traces (Arciuli & Simpson, 2012; Frank, Tenenbaum, & Gibson, 2013; Kóbor, Janacsek, Takács, & Nemeth, 2017), even after a substantial period of no exposure (e.g., 5 months; see Morgan-Short, Finger, Grey, & Ullman, 2012), which is essential for understanding how artificial language studies can scale up to real world applications.

Previous research suggests that retrieval practice, the act of spacing tests throughout the learning process, can be helpful for improving memory (for reviews, see Balota, Duchek, & Logan, 2007; Cepeda, Pashler, Vul, Wixted, & Rohrer, 2006). However, the amount of time between tests is also important, as some research suggests that younger adults benefit more from a short lag between tests relative to a longer lag (Maddox & Balota, 2015). In the current study, participants engaged in two retrieval attempts on Day 1 (immediately and 15 minutes after familiarization), followed by a longer delay of 1 week prior to the next retention test. It is possible that these repeated tests boosted retention, although only for the trained exemplars (Noun Test and Article Test) and not for generalization to new nouns. Previous research suggests that one retrieval attempt is sufficient to stimulate better long-term learning (for meta-analyses, see Adesope, Trevisan, & Sundararajan, 2017; Rowland, 2014). However, it remains an open question for future research as to whether it is more beneficial for long-term retention to test immediately or after a brief delay; testing shortly after introducing a novel concept could perhaps be particularly beneficial for L2 learning contexts.

### The Role of Individual Differences

Individual differences may also affect whether talker variability acts as a desirable difficulty. Previous research suggests that working memory is related to various aspects of vocabulary and grammar learning, for both first and second language learners (e.g., Martin & Ellis, 2012; Verhagen & Leseman, 2016). Thus, we included a working memory task that assesses online monitoring, updating, and manipulation (Owen, McMillan, Laird, & Bullmore, 2005) to identify possible individual differences that may affect performance on the task. We found that working memory was only related to word learning in Experiment 1, and individual differences in working memory capacity did not predict performance on any other aspects of learning.

Additionally, individual motivation to learn may be an important factor in eliciting desirable difficulties. For example, Suzuki et al. (2019) argue that, for L2 learning, desirable difficulties may in part be related to learner-specific factors such as motivation. They argue that “deliberate practice” (Ericsson, 2006), which should bring out the learner’s near-maximum effort by requiring full attention and taking place outside the comfort zone, may be a critically important variable to consider when investigating the contexts under which difficult learning conditions may be desirable. Thus, in our study we may not have found evidence that talker variability acted as a desirable difficulty if our learners did not in fact find the processing of talker variability sufficiently challenging. A measure of perceived difficulty or individual aptitude for contending with talker variability (Perrachione et al., 2011) could be helpful for future research. Further, the implicit (or, at least, incidental, as the participants were not told that they were being tested on grammatical relations) nature of the task used in the current study does not allow us to know whether participants used strategies, and if so which, in order to learn the components of the artificial grammar. Some participants could have been more deliberate than others in their approach to the task, trying to figure out the regularities. That is, talker variability may have different effects depending on the learning strategy (or lack thereof) that participants employ.

### Grammatical Dependencies

The category rule was designed to simulate, to some extent, grammatical gender, which some languages use to categorize nouns into subclasses based on linguistic or arbitrary criteria (Corbett, 1991). These languages often require gender agreement, matching the grammatical gender of the noun with other grammatical classes that carry gender markings, such as adjectives and articles (see Carstens, 2000; Grüter, Lew-Williams, & Fernald, 2012). Although some languages (e.g., Italian and Spanish) generally mark grammatical gender with a phonological rule (as in the current experiment), not all languages with grammatical gender have similarly transparent rule assignment. Further, across languages, assignment of gender is largely arbitrary (Corbett, 1991). Considering the arbitrary nature of this grammatical dependency, it is not surprising that it is notoriously difficult for L2 learners to acquire, particularly if their first language does not use grammatical gender (e.g., Dewaele & Véronique, 2001; Montrul, Foote, & Perpiñán, 2008; White, 2003).

Our findings provide an additional source of evidence that this type of grammatical dependency is difficult for monolingual English speakers to acquire. More generally, learners often struggle to acquire grammatical concepts that are not present in the native language via implicit learning mechanisms, as this study may have engendered. This difficulty has also been highlighted in studies using Brocanto2, a fully productive artificial language based on universal requirements for natural language, which also has gender agreement between articles, nouns, and adjectives (Morgan-Short, Sanz, Steinhauer, & Ullman, 2010, adapted from Friederici, Steinhauer, & Pfeifer, 2002; and see Pili-Moss, 2021, for a more recent study using an adapted Brocanto2 with child learners). Even after extensive practice, participants did not exhibit ceiling performance for discriminating between correct sentences and incorrect sentences in which gender agreement was violated (Morgan-Short et al., 2010). Thus, training over much longer periods of time may be particularly important for learning grammatical constructs that do not exist in the first language.

## Limitations

The artificial grammar used in these experiments was designed to afford learning of multiple aspects of grammar, so that we could explore whether difficulty in one aspect of learning (processing variability in the acoustic signal from different talkers) might yield benefits for others (learning vocabulary and grammatical relations). One limitation of this approach is that the artificial grammar was quite challenging for learners, as evidenced by a lack of generalization. As we expected a benefit for talker variability to be most evident for abstracting the category rule and generalizing to novel exemplars, the lack of generalizable knowledge limits the possible conclusions that can be drawn. Thus, it is possible that desirable difficulties may emerge only in certain contexts such as learning phonological contrasts. Another possible limitation is that there was insufficient variability to incur a processing cost, because the input in the current experiments was provided only by female speakers. For example, there was a greater total number of talkers and a potentially wider range of acoustic variability in experiments that have reported processing costs for adult learners, such as in the Mullennix et al. (1989) study, which used seven male and eight female talkers.

## Conclusions and Future Directions

In two experiments, we demonstrated that high and limited talker variability can differentially impact learning to some extent but that neither type of variability acted as a desirable difficulty in the context of artificial grammar learning. Nevertheless, as we noted in the rationale for the study, talker variability can impose costs under some conditions and benefits in others. Although our research did not delineate a relationship between these patterns, the issue is still worth pursuing. Future research should explore manipulations that can enhance learning and generalization in the talker variability paradigm. For example, increasing the length of exposure may allow participants more time to learn a nonadjacent category rule. Alternatively, adding a communicative aspect to the task to increase motivation, such as in studies using the interactive computer game Brocanto2 (e.g., Friederici et al., 2002), may lead to more engaged effort by the participants. A related avenue to explore is whether learning from different talkers interacts with the training and/or testing tasks, such as the direction of testing (from the concept or the L1 words to the new language, or vice versa) or different types of form overlap as suggested by Kemp and McDonald (2021). Lastly, future research should also investigate whether the age of the learner and their previous language experience are important factors in how acoustic variability influences learning.

## Supplementary Material

Supporting materials **Appendix S1.** Stimuli Used in Experiments 1 and 2.
**Appendix S2.** Design Counterbalancing Table.
**Appendix S3.** Mean Pitch for Experimental Talkers.
**Appendix S4.** Experiment 1 and Experiment 2 Model Tables.
**Appendix S5.** Final Vowel Discrimination for Experiment 1 Stimuli.

## Figures and Tables

**Table 1:** Experiment 1: Means, standard deviations, estimated marginal means (*EMM*), and 95% confidence intervals for each test type across test time and talker condition

	Immediate		Short delay		Long delay	
Talker condition	*M(SD)* *EMM* [95% Cl]	*t*(47) *P*	*M(SD)* *EMM* [95% Cl]	*t*(47) *P*	*M(SD)* *EMM* [95% Cl]	*t*(47) *P*
Noun Test						
1-Talker	80.0 (16)	13.0	81.1 (14)	15.6	71.2 (20)	7.43
	84 [78, 89]	**<.001**	85 [79, 90]	**<.001**	75 [67, 82]	**<.001**
2-Talker	75.7 (18)	10.1	75.9 (16)	11.2	69.3 (17.5)	7.61
	80 [73, 86]	**<.001**	80 [73, 86]	**<.001**	73 [65, 80]	**<.001**
8-Talker	77.4 (18)	10.8	76.2 (20)	9.27	67.5 (20)	6.22
	82 [75, 88]	**<.001**	81 [74, 87]	**<.001**	72 [63, 80]	**<.001**
Article Test						
1-Talker	59.2 (15)	4.13	57.6 (14)	3.82	58.7 (16)	4.37
	60 [54, 64]	**<.001**	58 [53, 63]	**<.001**	59 [54, 64]	**<.001**
2-Talker	51.0 (20)	0.36	51.0 (18)	0.4	50.7 (21)	0.23
	51 [44, 58]	.71	51 [44, 58]	.68	51 [44, 58]	.82
8-Talker	60.6 (15)	4.81	61.5 (13)	6.03	57.8 (16)	3.39
	61 [55, 66]	**<.001**	62 [56, 67]	**<.001**	58 [53, 63]	**.001**
Generalization Test						
1-Talker	52.1 (17)	0.87	46.4 (15)	1.67	47.4 (17)	1.12
	52 [48, 56]	.38	46 [42, 51]	.10	47 [43, 52]	.27
2-Talker	53.6 (14)	1.78	47.4 (16)	1.12	50.9 (17)	0.36
	53 [49, 57]	.08	47 [43, 52]	.27	52 [47, 56]	.72
8-Talker	50.1 (20)	0.06	54.7 (16)	1.99	47.5 (17)	0.97
	50 [46, 55]	.95	55 [51, 59]	.05	48 [43, 52]	.33

*Note*. *T* tests reflect comparison against chance (50%) performance. **Boldface** denotes above-chance performance after Bonferroni correction for nine multiple comparisons: Corrected *α*= .006.

**Table 2:** Experiment 2: Means, standard deviations, estimated marginal means (*EMM*), and 95% confidence intervals for test type across test time and talker condition

	Immediate		Short delay		Long delay	
Talker condition	*M(SD)* *EMM* [95% Cl]	*t* ^a^ *p*	*M(SD)* *EMM* [95% Cl]	*t* ^a^ *p*	*M(SD)* *EMM* [95% Cl]	*t* ^a^ *p*
Noun Test						
1-Talker	77.1 (18)	10.48	77.1 (16)	11.87	71.5 (17)	8.91
	80 [73, 86]	**<.001**	80 [73, 86]	**<.001**	75 [66, 82]	**<.001**
2-Talker	75.2 (17)	10.17	73.8 (18)	8.85	67.4 (19)	6.24
	79 [71, 86]	**<.001**	78 [69, 84]	**<.001**	71 [61, 79]	**<.001**
8-Talker	75.3 (18)	10.0	74.0 (18)	9.52	69.1 (16)	8.40
	79 [72, 85]	**<.001**	78 [70, 84]	**<.001**	73 [64, 80]	**<.001**
Article Test						
1-Talker	61.0 (12)	6.34	61.6 (15)	5.39	59.2 (18)	3.56
	61 [56, 67]	**<.001**	62 [56, 67]	**<.001**	59 [53,64]	**.002**
2-Talker	65.4 (13)	8.44	60.8 (16)	4.57	56.6 (23)	1.96
	66 [61, 71]	**<.001**	61 [56, 66]	**<.001**	57 [51, 62]	.06
8-Talker	64.9 (13)	8.38	60.9 (11)	7.22	60.1 (14)	5.19
	66 [60, 70]	**<.001**	62 [56, 67]	**<.001**	61 [55, 66]	**<.001**
Generalization Test						
1-Talker	51.9 (14)	0.93	45.1 (16)	2.15	51.9 (16)	.85
	52 [48, 56]	.35	45 [41, 49]	.04	52 [48, 56]	.40
2-Talker	49.1 (15)	0.41	51.9 (16)	0.83	54.4 (16)	1.93
	49 [45, 53]	.68	52 [47, 56]	.40	54 [50, 58]	.06
8-Talker	51.9 (14)	0.96	50.5 (15)	0.24	49.5 (15)	0.23
	52 [48, 56]	.34	51 [48, 56]	.81	50 [46, 54]	.81

*Note*. *T* tests reflect comparison against chance (50%) performance. **Boldface** denotes above-chance performance after Bonferroni correction for nine multiple comparisons: corrected *α*= .006.

a 1-Talker: *df* = 47; 2-Talker: *df* = 46; 8-Talker: *df* = 51.

## Does the Variability Between Different Talkers Help or Hinder Grammar Learning?

### What This Research Was AboutandWhy It Is Important

One of the challenges confronting language learners is that speech production is highly variable, both within and across individuals. Learners must ignore some differences (e.g., different pronunciations of the same word) in order to attend to relevant features (such as changes in sound that indicate different words). Understanding how learners overcome the challenge of dealing with multiple talkers has implications for understanding the process of language acquisition and for identifying language deficits and improving instruction. This study investigated whether learning from different talkers can be *both* challenging and yet benefit language learning, a characteristic called “desirable difficulty” that has been evidenced in other areas of cognitive processing. We were particularly interested in: (1) how many talkers participants learned from and (2) whether learning was retained over time.

### What the Researchers Did

- In two similar related experiments, college-aged participants learned a small artificial language consisting of names for two categories of novel objects.
- Each category of objects was paired with one article before it (sem or bol) that was specific to that category of objects.
- In Experiment 1 the sounds at the end of the words (i.e., the suffixes) were very similar across the two categories (difficult to distinguish from each other). In Experiment 2, the ends of the words were distinct across categories (easier to distinguish).
- Participants could “infer” (work out) the grammatical relationship between the specific article and the sound at the end of the words, but they were not informed about the grammatical relationship directly.
- Participants learned the new words and articles from either one, two, or eight talkers.
- Participants were tested on: (i) the names of the objects, (ii) which article matched, and (iii) if they could generalize the grammatical relationship between the article and the word endings to new nouns (that were not presented during training).
- Participants were tested at three time points: immediately after learning, after a short delay, and after 1 week.

### What the Researchers Found

- Participants who learned from one talker and eight talkers demonstrated learning of both the names for the objects and the articles, in both Experiments 1 and 2.
- Participants who learned from two talkers demonstrated learning of only the names of the objects in Experiment 1, but both the names of the objects and the articles in Experiment 2.
- However, participants were not able to generalize the relationship between the articles and suffixes to new nouns, regardless of how many talkers they learned from or how similar the suffixes were across the different categories of nouns.
- High talker variability (eight talkers) did not interfere with learning, but also did not lead to benefits, while limited talker variability (two talkers) interfered with learning only under more difficult conditions (where the ends of words were similar).
- Learning was robust at all time points and retained even after the delay of 1 week.

### Things to Consider

- One possible limitation of the current study is that participants did not have enough time to learn to generalize the grammatical relationship between the specific article and the sound at the end of the word.
- Talker variability may be relevant for learning some aspects of language (e.g., sounds) but not others (e.g., grammar).
- Future research will investigate how talker variability may differentially impact learning across stages of development.

## Footnotes

1 All participants received credit for the parts of the study they completed, regardless of the effort rating they provided. Effort rating did not differ across conditions: one-talker, 8.41; two-talker, 8.27; eight-talker, 8.55.

2 Because each test trial of the artificial grammar tested knowledge of a specific item, reliability indices are difficult to interpret (i.e., we cannot expect that all participants will learn the same word-object labels). Nonetheless, they are reported here for full transparency. Internal consistency (Cronbach alpha) ranged between .48 – .57 (Experiment 1) and between .47 – .57 (Experiment 2) for the Noun Test; between .29 – .42 (Experiment 1) and between .26 – .45 (Experiment 2) for the Article Test; and between .35 – .56 (Experiment 1) and between .22 – .51 (Experiment 2) for the Generalization Test.

3 We investigated whether the addition of an interaction between talker variability condition and N-back would improve model fit. Model comparisons revealed that including these interactions did not improve model fit.

4 Models were optimized using the “bobyqa” optimizer, because they did not converge otherwise. The maximal random-effects structure that converged for most models was (1 + TalkerVariabilityCondition|Subject) + (1|Item). For two of the models (Experiment 2 Article and Experiment 1 Generalization), this did not converge, so the random-effects structure was (1|Subject) + (1|Image) and (1|Subject), respectively.

5 Effort rating for Experiment 2 did not differ across conditions: one-talker, 8.12; two-talker, 8.21; eight-talker, 8.33.
