# Supplementary material for: Desirable Difficulties in Language Learning? How Talker Variability Impacts Artificial Grammar Learning
Source: Lang Learn. Author manuscript; Available in PMC 2022 Dec 1. (PMC8945865; doi:10.1111/lang.12464)
Supplement: Supporting materials — Appendix S1. Stimuli Used in Experiments 1 and 2. Appendix S2. Design Counterbalancing Table. Appendix S3. Mean Pitch for Experimental Talkers. Appendix S4. Experiment 1 and Experiment 2 Model Tables. Appendix S5. Final Vowel Discrimination for Experiment 1 Stimuli. [file NIHMS1734872-supplement-Supporting_materials.pdf]

Supporting information for: Bulgarelli, F., & Weiss, D. J. Desirable difficulties in language learning? How talker variability impacts artificial grammar learning. Article accepted in *Language Learning* on 2 March 2021.

Appendix S1: Stimuli Used in Experiments 1 and 2

Table S1 Novel objects and nonce words used in Experiments 1 and 2

Category 1				Category 2			
Object	Label	Object	Label	Object	Label	Object	Label
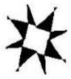	E1:Gotca<br>E2:Gotki	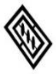	E1:Tarrena<br>E2:Tarreni	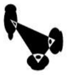	Pilkaw	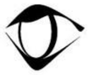	Pangalaw
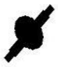	E1:Peadla<br>E2:Peadli	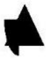	E1:Klorda<br>E2:Klordi	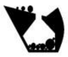	Tenderaw	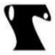	Haseraw
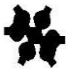	E1:Prittera<br>E2:Pritteri	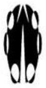	E1:Sooba<br>E2:Soobi	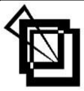	Higgeraw	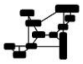	Daintaw
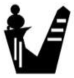	E1:Fispa<br>E2:Fispi	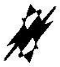	E1:Grintera<br>E2:Grinteri	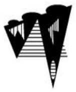	Foungeraw	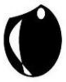	Zibaw
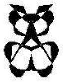	E1:Veama<br>E2:Veami	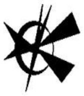	E1:Tifa<br>E2:Tifi	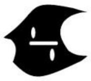	Drockaw	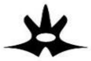	Naretaw
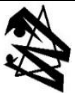	E1:Feecha<br>E2:Feechi	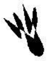	E1:Barcha<br>E2:Barchi	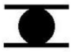	Bosaw	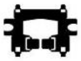	Koobaw
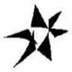	E1:Glinka<br>E2:Glinki	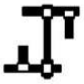	E1:Chosta<br>E2:Chosti	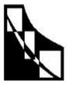	Tallotaw	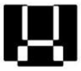	Pluntaw
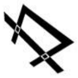	E1:Durroba<br>E2:Durrobi	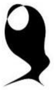	E1:Patcheta<br>E2:Patcheti	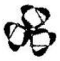	Cheemaw	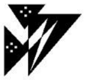	Jefaw
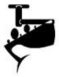	E1:Lummora<br>E2:Lummori	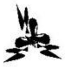	E1:Tolara<br>E2:Tolari	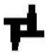	Perminaw	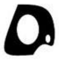	Gipaw
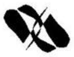	E1:Dulcha<br>E2:Dulchi	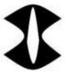	E1:Bema<br>E2:Bemi	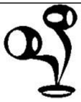	Theckeraw	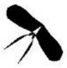	Massettaw

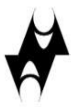	E1:Sarna<br>E2:Sarni	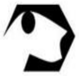	E1:Suncha<br>E2:Sunchi	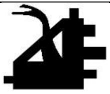	Kitaw	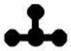	Tomaw
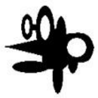	E1:Vaba<br>E2:Vabi	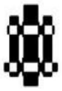	E1:Pedlina<br>E2:Pedlini	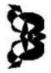	Plockaw	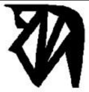	Blepaw
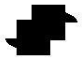	E1:Scowna<br>E2:Scowni	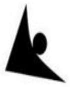	E1: Briskala<br>E2: Briskali	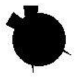	Doffaw	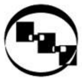	Franaw
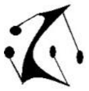	E1:Poska<br>E2:Poski	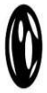	E1:Narbeda<br>E2:Narbedi	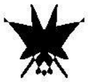	Cridaw	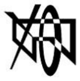	Cornicka<br>w
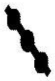	E1:Parioda<br>E2:Pariodi	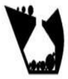	E1: Binka<br>E2: Benki	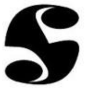	Murleraw	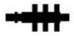	Glockaw

Note. Category 1 objects ended in /-a/ in Experiment 1 (E1) and in /-i/ in Experiment 2 (E2), both options are listed here.

Appendix S2: Design Counterbalancing Table

Table S2 Design counterbalancing table: Talkers used in each familiarization and test condition

Condition	1-Talker		2-Talker		8-Talker	
Familiarization	LK	TW	LK + TW	LK + TW	All 8 (LK, TW, MP, RF, NS, SW, KM, BS)	All 8 (LK, TW, MP, RF, NS, SW, KM, BS)
Test	LK	TW	LK	TW	LK	TW

Appendix S3: Mean Pitch for Experimental Talkers

We measured pitch (F0) for each word recorded by every speaker in order to provide some measure of cross-speaker acoustic differences. Tables S3.1 and S3.2 displays the means and standard deviations for each speaker (identified by their initials), for each experiment. The bolded speakers are those used during the test phase in all conditions.

Table S3.1 Means and standard deviations of pitch in Hertz for stimuli recorded by each speaker in Experiment 1

Speaker	**LK**	**TW**	MP	RF	NS	SW	KM	BS
M	199.84	177.17	183.46	179.53	169.74	199.71	228.80	261.01
(*SD*)	(20.50)	(25.30)	(31.48)	(21.69)	(16.08)	(16.85)	(22.48)	(24.46)

Note. Bolded speakers were used in all testing phases.

Table S3.2 Means and standard deviations of pitch in Hertz for stimuli recorded by each speaker in Experiment 2

Speaker	**LK**	**TW**	MP	RF	NS	SW	KM	BS
M	217.15	184.56	193.29	182.96	168.34	199.24	226.81	267.31
(*SD*)	(13.13)	(18.69)	(21.93)	(11.31)	(13.27)	(9.07)	(16.29)	(23.55)

Note. Bolded speakers were used in all testing phases.

Appendix S4: Experiment 1 and Experiment 2 Model Tables

Table S4.1 Fixed effects for Experiment 1: Noun Test

Term	*b*	*SE*	95% CI	*z*	*p*
(Intercept)	–0.30	0.59	[-1.44, .85]	–0.51	.61
1-Talker/2&8-Talker	0.20	0.18	[-.15, .56]	1.13	.26
2-Talker/8-Talker	–0.05	0.22	[-.47, .38]	–0.22	.83
Immediate/ShortDelay	–0.01	0.09	[-.18, .17]	–0.06	.95
Immediate&ShortDelay/LongDelay	–0.52	0.07	[-.66, -.38]	–7.19	.00
Nback	1.75	0.60	[.58, 1.91]	2.93	.00
TestTalker	0.14	0.17	[-.20, .48]	0.83	.41
1-Talker/2&8-Talker × Immediate/ShortDelay	–0.10	0.19	[-.48, .27]	–0.55	.58
2-Talker/8-Talker × Immediate/ShortDelay	–0.10	0.21	[-.52, .31]	–0.49	.62
1-Talker/2&8-Talker × Immediate&ShortDelay/LongDelay	–0.12	0.15	[-.42, .18]	–0.76	.45
2-Talker/8-Talker × Immediate&ShortDelay/LongDelay	0.17	0.17	[-.17, .51]	0.98	.33

Table S4.2 Random effects for Experiment 1: Noun Test

Group	Term	σ^2
Subject	sd__(Intercept)	0.83
Subject	cor__(Intercept).1-Talker/2&8-Talker	–0.14
Subject	cor__(Intercept).2-Talker/8-Talker	–0.27
Subject	sd__1-Talker/2&8-Talker	0.53
Subject	cor__1-Talker/2&8-Talker.2-Talker/8-Talker	–0.18
Subject	sd__2-Talker/8-Talker	0.69
Image	sd__(Intercept)	0.43

Table S4.3 Fixed effects for Experiment 1: Article Test

Term	*b*	*SE*	95% CI	*z*	*p*
(Intercept)	0.04	0.29	[-.62, .55]	0.13	.90
1-Talker/2&8-Talker	0.12	0.08	[-.06, .31]	1.42	.15
2-Talker/8-Talker	–0.38	0.12	[-.60, -.17]	–3.04	.00
Immediate/ShortDelay	0.01	0.07	[-.13, .15]	0.09	.92
Immediate&ShortDelay/LongDelay	–0.04	0.06	[-.16, .08]	–0.67	.50
Nback	0.31	0.29	[-.21, .99]	1.08	.28
TestTalker	–0.01	0.08	[-.18, .17]	–0.21	.83
1-Talker/2&8-Talker × Immediate/ShortDelay	0.09	0.15	-.20, .38]	0.59	.55
2-Talker/8-Talker × Immediate/ShortDelay	0.03	0.18	[-.31, .37]	0.17	.86
1-Talker/2&8-Talker × Immediate&ShortDelay/LongDelay	0.08	0.13	[-.18, .33]	0.60	.55
2-Talker/8-Talker × Immediate&ShortDelay/LongDelay	0.14	0.15	[-.15, .44]	0.94	.35

Table S4.4 Random effects for Experiment 1: Article Test

Group	Term	σ^2
Subject	sd__(Intercept)	0.19
Subject	cor__(Intercept).1-Talker/2&8-Talker	–0.76
Subject	cor__(Intercept).2-Talker/8-Talker	0.50
Subject	sd__1-Talker/2&8-Talker	0.42
Subject	cor__1-Talker/2&8-Talker.2-Talker/8-Talker	–0.77
Subject	sd__2-Talker/8-Talker	0.76
Image	sd__(Intercept)	0.21

Table S4.5 Fixed effects for Experiment 1: Generalization Test

Term	*b*	*SE*	95% CI	*z*	*p*
(Intercept)	–0.35	0.19	[-.73, .03]	–1.82	.07
1-Talker/2&8-Talker	–0.09	0.06	[-.21, .03]	–1.43	.15
2-Talker/8-Talker	–0.01	0.07	[-.15, .13]	–0.12	.91
Immediate/ShortDelay	0.09	0.07	[-.04, .23]	1.33	.18
Immediate&ShortDelay/LongDelay	–0.07	0.06	[-.19, .04]	–1.21	.23
Nback	0.33	0.20	[-.06, .73]	1.65	.10
TestTalker	0.05	0.06	[-.06, .17]	0.88	.38
1-Talker/2&8-Talker × Immediate/ShortDelay	0.21	0.15	[-.08, .49]	1.43	.15
2-Talker/8-Talker × Immediate/ShortDelay	0.41	0.17	[.08, .74]	2.44	.01
1-Talker/2&8-Talker × Immediate&ShortDelay/LongDelay	0.00	0.13	[-.25, .25]	0.00	1.00
2-Talker/8-Talker × Immediate&ShortDelay/LongDelay	0.25	0.15	[-.04, .53]	1.69	.09

Table S4.6 Random effects for Experiment 1: Generalization Test

Group	Term	σ^2
Subject	sd__(Intercept)	0.11
Image	sd__(Intercept)	0.03

Table S4.7 Fixed effects for Experiment 2: Noun Test

Term	*b*	*SE*	95% CI	*z*	*p*
(Intercept)	0.48	0.47	[-.43, 1.40]	1.04	.30
1-Talker/2&8-Talker	0.12	0.17	[-.21, .45]	0.70	.48
2-Talker/8-Talker	–0.03	0.20	[-.43, .36]	–0.17	.86
Immediate/ShortDelay	0.05	0.08	[-.11, .22]	0.61	.54
Immediate&ShortDelay/LongDelay	–0.36	0.07	[-.49, -.22]	–5.09	.00
Nback	0.91	0.49	[-.04, 1.86]	1.87	.06
TestTalker	–0.01	0.16	[-.33, .32]	–0.03	.98
1-Talker/2&8-Talker × Immediate/ShortDelay	–0.09	0.18	[-.45, .26]	–0.52	.60
2-Talker/8-Talker × Immediate/ShortDelay	0.01	0.20	[-.39, .40]	0.04	.97
1-Talker/2&8-Talker × Immediate&ShortDelay/LongDelay	0.03	0.15	[-.26, .33]	0.22	.83
2-Talker/8-Talker × Immediate&ShortDelay/LongDelay	–0.08	0.17	[-.41, .25]	–0.49	.62

Table S4.8 Random effects for Experiment 2: Noun Test

Group	Term	σ^2
Subject	sd__(Intercept)	0.64
Subject	cor__(Intercept).1-Talker/2&8-Talker	–0.13
Subject	cor__(Intercept).2-Talker/8-Talker	0.11
Subject	sd__1-Talker/2&8-Talker	0.89
Subject	cor__1-Talker/2&8-Talker.2-Talker/8-Talker	–0.15
Subject	sd__2-Talker/8-Talker	0.95
Image	sd__(Intercept)	0.48

Table S4.9 Fixed effects for Experiment 2: Article Test

Term	*b*	*SE*	95% CI	*z*	*p*
(Intercept)	0.18	0.24	[-.29, .65]	0.77	.44
1-Talker/2&8-Talker	–0.05	0.09	[-.22, .12]	–0.59	.55
2-Talker/8-Talker	–0.05	0.10	[-.25, .14]	–0.54	.59
Immediate/ShortDelay	0.12	0.07	-.02, .26]	1.72	.08
Immediate&ShortDelay/LongDelay	–0.17	0.06	[-.29, -.05]	–2.83	.00
Nback	0.29	0.26	[-.21, .79]	1.13	.26
TestTalker	0.03	0.08	[-.13, .19]	0.42	.67
1-Talker/2&8-Talker × Immediate/ShortDelay	–0.21	0.15	[-.51, .09]	–1.37	.17
2-Talker/8-Talker × Immediate/ShortDelay	0.03	0.17	[-.31, .37]	0.17	.87
1-Talker/2&8-Talker × Immediate&ShortDelay/LongDelay	0.10	0.13	[-.16, .35]	0.75	.46
2-Talker/8-Talker × Immediate&ShortDelay/LongDelay	–0.16	0.15	[-.45, .13]	–1.10	.27

Table S4.10 Random effects for Experiment 2: Article Test

Group	Term	σ^2
Subject	sd__(Intercept)	0.34
Image	sd__(Intercept)	0.20

Table S4.11 Fixed effects for Experiment 2: Generalization Test

Term	*b*	*SE*	95% CI	*z*	*p*
(Intercept)	–0.07	0.16	[-.39, .24]	–0.46	.65
1-Talker/2&8-Talker	–0.07	0.06	[-.18, .05]	–1.13	.26
2-Talker/8-Talker	0.04	0.07	[-.10, .17]	0.55	.58
Immediate/ShortDelay	0.07	0.07	[-.06, .20]	1.06	.29
Immediate&ShortDelay/LongDelay	0.08	0.06	[-.04, .19]	1.31	.19
Nback	0.18	0.18	[-.16, .53]	1.05	.30
TestTalker	–0.03	0.06	[-.14, .08]	–0.60	.55
1-Talker/2&8-Talker × Immediate/ShortDelay	0.30	0.15	[.01, .58]	2.06	.04
2-Talker/8-Talker × Immediate/ShortDelay	–0.17	0.16	[-.49, .15]	–1.04	.30
1-Talker/2&8-Talker × Immediate&ShortDelay/LongDelay	0.10	0.13	[-.15, .34]	0.78	.44
2-Talker/8-Talker × Immediate&ShortDelay/LongDelay	0.22	0.14	[-.06, .50]	1.57	.12

Table S4.12 Random effects for Experiment 2: Generalization Test

Group	Term	σ^2
Subject	sd__(Intercept)	0.04

Appendix S5. Final Vowel Discrimination for Experiment 1 Stimuli

Participants in Experiment 1 exhibited difficulty generalizing to novel exemplars. It is possible that participants did not learn to generalize to novel exemplars because they could not hear the difference in the vowels at the end of the words that served to differentiate the two categories. We investigated this possibility by testing whether a different group of participants could distinguish between the vowels at the end of words when the words were presented in isolation. Participants were presented with pairs of words that either ended in the same vowel or in different vowels and were instructed to identify whether the final vowel was the same or different across the two words.

S5.1 Participants

Participants were 177 native English speakers (M_{age} = 18.93 years, SD = 1.30, 29 males) recruited from the Psychology Subject Pool. All participants completed another unrelated experiment in the laboratory prior to completing this task.

S5.2 Stimuli and procedure

Participants were seated in a sound attenuated booth and instructed that they would be hearing two words separated by a short pause. Their task would be to listen carefully to the sounds that occurred at the end of the words, and after hearing both tokens, to decide whether they were same or different. There was no time limit for responses. On half of the trials, participants heard two words from the same category ending in the same vowel, and, on the remaining trials, participants heard words from different categories ending in different vowels. Participants completed 28 trials in total. For each participant, all stimuli were presented by a single talker, and the talker was counterbalanced across participants.

S5.3 Results

Planned *t* tests comparing performance to chance levels revealed that participants could accurately determine whether the vowel at the end of a pair of words was the same or different (all *p*s < .01). As Table S5.1 shows, there was variability in accuracy on this task across talkers, though performance for all talkers was above chance. Note that the difference between speakers LK and TW (which were used for all tests, and in the 1- and 2-Speaker conditions) was not significant, $t(41.98) = .017$, $p = .99$.

Table S5.1

Speaker	Accuracy	Significance
LK	20.15 (2.99)	$t(19) = 9.18$, $p < .001$
TW	20.17 (3.53)	$t(23) = 8.54$, $p < .001$
MP	16.52 (2.71)	$t(20) = 4.26$, $p < .001$
RF	17.81 (2.72)	$t(21) = 6.59$, $p < .001$
NS	16.50 (2.54)	$t(23) = 4.83$, $p < .001$
SW	15.71 (2.19)	$t(20) = 3.58$, $p = .002$
KM	18.73 (2.86)	$t(21) = 7.74$, $p < .001$
BS	20.22 (2.92)	$t(22) =10.20$, $p < .001$

S5.4 Discussion.

In sum, while there was variability in accuracy across talkers, participants performed significantly above chance on this task. Importantly, the two talkers used during all tests produced vowels that were most distinguishable relative to some of the other talkers, and thus performance on the tests should not have been impacted by differences in clarity across talker. Further, the results highlight the naturalistic variability presented to participants in the

experiment, because there is inherent variability in the way individuals produce phonemes (Dorman, Studder-Kennedy, & Raphael, 1977; Peterson & Barney, 1952).

Performance on this task was also consistent with other phoneme discrimination tasks with difficult phoneme contrasts where average or peak performance was rarely above 75% (Maye & Gerken, 2000, 2001). Compared to these other phoneme discrimination studies, the current vowel discrimination paradigm was arguably more difficult because participants were asked to discriminate between words that are not minimal pairs and thus differ in more than just the target phoneme. Due to the process of coarticulation, having different word frames further changes how each vowel is instantiated (Liberman, Coopers, Shankweiler, & Studdert-Kennedy, 1967). Therefore, this follow-up revealed that participants can distinguish between the two vowels at above chance levels but also highlighted the difficult nature of the experimental task and provided further justification for Experiment 2 in which participants were presented with an easier vowel contrast.

References

Dorman, M. F., Studdert-Kennedy, M., & Raphael, L. J. (1977). Stop-consonant recognition: Release bursts and formant transitions as functionally equivalent, context-dependent cues. *Perception & Psychophysics, 22*, 109–122. https://doi.org/10.3758/BF03198744

Liberman, A. M., Coopers, F. S., Shankweiler, D. P., & Studdert-Kennedy, M. (1967). Perception of the speech code. *Psychological Review*, *74*, 431–461. https://doi.org/10.1037/h0020279

Maye, J., & Gerken, L. (2000). Learning phonemes without minimal pairs. In S. C. Howell, S. A. Fish, & T. Keith-Lucas (Eds.), *Proceedings of the 24th annual Boston University Conference on Language Development* (pp. 522–533). Somerville, MA: Cascadilla.

http://citeseerx.ist.psu.edu/viewdoc/download?doi=10.1.1.188.5940&rep=rep1&type=pdf

Maye, J., & Gerken, L. (2001). Learning phonemes : How far can the input take us ? In A. H.-J. Do, L. Domínguez, & A. Johansen (Eds.), Proceedings of the *25th annual Boston University Conference on Language Development* (pp, 480–490). Somerville, MA: Cascadilla

Peterson, G. E., & Barney, H. L. (1952). Control methods used in a study of the vowels. *The Journal of the Acoustical Society of America*, *24*, 175–184. https://doi.org/10.1121/1.1906875
